# Identifying genes for neurobehavioural traits in rodents: progress and pitfalls

**DOI:** 10.1242/dmm.027789

**Published:** 2017-04-01

**Authors:** Amelie Baud, Jonathan Flint

**Affiliations:** 1 European Molecular Biology Laboratory, European Bioinformatics Institute, Wellcome Genome Campus, Hinxton, Cambridge CB10 1SD, UK; 2 Center for Neurobehavioral Genetics, Semel Institute for Neuroscience and Human Behavior, University of California, Los Angeles, Los Angeles, CA 90095-1761, USA

**Keywords:** Genetics of behaviour, Quantitative trait loci, Rodent resources

## Abstract

Identifying genes and pathways that contribute to differences in neurobehavioural traits is a key goal in psychiatric research. Despite considerable success in identifying quantitative trait loci (QTLs) associated with behaviour in laboratory rodents, pinpointing the causal variants and genes is more challenging. For a long time, the main obstacle was the size of QTLs, which could encompass tens if not hundreds of genes. However, recent studies have exploited mouse and rat resources that allow mapping of phenotypes to narrow intervals, encompassing only a few genes. Here, we review these studies, showcase the rodent resources they have used and highlight the insights into neurobehavioural traits provided to date. We discuss what we see as the biggest challenge in the field – translating QTLs into biological knowledge by experimentally validating and functionally characterizing candidate genes – and propose that the CRISPR/Cas genome-editing system holds the key to overcoming this obstacle. Finally, we challenge traditional views on inbred versus outbred resources in the light of recent resource and technology developments.

## Abstract (precis)

**Summary:** This Review discusses the rodent resources that exist to map neurobehavioural traits at high resolution and identify causal variants and genes.

## Introduction

According to a World Health Organization (WHO) survey of 14 countries in the Americas, Europe, the Middle East, Africa and Asia (Demyttenaere et al., 2004), psychiatric disorders are relatively common worldwide, although the overall prevalence varies widely by country. Anxiety disorders are the most common disorders, followed by mood, substance and impulse-control disorders. Importantly, psychiatric disorders are typically associated with impairment greater than that due to serious chronic physical diseases (Demyttenaere et al., 2004).

Despite the high prevalence of psychiatric disorders and the important individual and societal burden, relatively little is known about the underlying biological mechanisms. As a result, diagnostic categories such as those of the widely influential Diagnostic and Statistical Manual of Mental Disorders (American Psychiatric Association, 2000) and International Statistical Classification of Diseases and Related Health Problems (World Health Organization, 2004) are defined based on symptoms (e.g. guilty ruminations, suicidal thoughts, low mood) and signs (e.g. impaired cognitive function, rapid speech).

Although such classification has enabled more reproducible diagnoses and better clinical management, it does not ensure that the diagnostic categories correspond to shared underlying causes and mechanisms (Owen, 2014). For example, major depressive disorder (MDD) is diagnosed if an individual presents with five out of nine symptoms, one of which must be depressed mood or loss of pleasure. Symptomatic heterogeneity suggests that psychiatric disorders are likely to stem from various causes and mechanisms (Casey et al., 2013). The same symptoms can also apply to different diagnostic categories (Allardyce et al., 2007) so that, in addition to heterogeneity within a disorder, common aetiologies between disorders are likely.

Research has provided evidence that the match between diagnostic categories and genetic causes is relatively poor. For example, genetic heterogeneity in MDD is evident from the poor genetic correlation between sexes [0.60, which is similar to the genetic correlation between MDD and bipolar disorder (BPD) at 0.64] (Flint and Kendler, 2014). Furthermore, several studies have provided evidence for a shared genetic component across diagnostic categories, including schizophrenia (SZ) and BPD (Lichtenstein et al., 2009; Purcell et al., 2009; Cross-Disorder Group of the Psychiatric Genomics Consortium, 2013a), SZ and MDD, BPD and MDD, attention-deficit/hyperactivity disorder (ADHD) and MDD, and SZ and autism spectrum disorders (ASD) (Cross-Disorder Group of the Psychiatric Genomics Consortium, 2013a). Genome-wide association studies (GWAS; see Glossary, Box 1) have also identified individual genetic variants that contribute to multiple psychiatric disorders (Psychiatric GWAS Consortium and Bipolar Disorder Working Group, 2011; Cross-Disorder Group of the Psychiatric Genomics Consortium, 2013b; Sullivan et al., 2012).

A further confounding factor is that healthy individuals may experience ‘hallmark’ symptoms of psychiatric disorders, such as delusions and hallucinations (Allardyce et al., 2007). Similarly, quantitative traits that are extreme in affected individuals vary substantially among healthy individuals (e.g. capacity for social communication is impaired in ASD but varies in the general population, Robinson et al., 2016). Importantly, variation of the relevant traits in healthy and affected individuals arises from similar genetic variants (Robinson et al., 2016; Lencz et al., 2014), suggesting that psychiatric disease is likely to be the extreme of a continuum rather than a discrete entity.

Therefore, there has been a push to reconsider the way in which research on psychiatric disorders is carried out and to stop focusing on current clinical diagnostic categories. Championing that vision, the American National Institute of Mental Health (NIMH) launched the Research Domain Criteria (RDoC) project (Insel et al., 2010), which encourages researchers to use a wider range of, preferably quantitative, measures related to mental health, including genetic, molecular, cellular, circuit-level and individual-level measures, as well as the family environment and social context, and to study the full spectrum of these variables in affected individuals and the general population (Insel et al., 2010; Casey et al., 2013). The goal of RDoC is that the classification of psychiatric disorders will, in the future, be informed by findings from neuroscience and genomics, and match aetiological processes. Furthermore, knowledge of the biological processes underlying psychiatric disorders would allow biological and behavioural tests to be used for diagnosis, prevention and treatment.

With increasing focus on specific aspects of psychiatric disorders rather than diagnostic categories, the importance of animal models in psychiatric research is evident (Markou et al., 2009). Many measures collected in humans to assess behavioural traits can be readily collected in rodents (e.g. neuroimaging, startle test, biochemical measurements), and measures that can only be collected in animal models for practical or ethical reasons (e.g. gene expression in the brain, response to psychosocial stress) may also contribute to a better understanding of neurobehavioural processes.

Of course, the way phenotypic variation arises will be important when considering the relevance of animal models to psychiatric research. Because most common psychiatric disorders have been shown to arise from a large number of genetic variants and are considered to be complex traits (see Glossary, Box 1), populations of mice and rats that segregate a large number of naturally occurring variants and present continuous phenotypic variation will be most relevant.

In this Review, we highlight rodent resources that have shed light on the genetic basis of behaviour and associated neurophysiological traits. More specifically, we focus on the outcome that we believe is of greatest interest to geneticists with a strong focus on disease, namely the identification of genes associated with neurobehavioural variation. Once genes have been associated with a trait, the pathways, cell types and neural circuits that are involved can be inferred, and potential therapeutic targets identified.

Because of our focus on complex behaviours and traits, we will not discuss animal models created to assess the role of one gene in isolation; instead, we will review rodent resources in which a large number of genetic variants segregate and give rise to phenotypic variation (for a review of both strategies and how they complement each other, see Williams and Auwerx, 2015).

The same resources can be used for the study of behaviours as for other complex traits. Indeed, although behaviours are ‘noisier’ phenotypes than average i.e. their heritability (see Glossary, Box 1) is slightly lower than that of other complex traits both in mice (Valdar et al., 2006b; Nicod et al., 2016; Parker et al., 2016) and rats (Baud et al., 2014b; Rat Genome Sequencing and Mapping Consortium, 2013), the effect sizes of quantitative trait loci (QTLs; see Glossary, Box 1) are not significantly lower (Flint and Mackay, 2009; Flint and Mott, 2008) or only very slightly lower (Nicod et al., 2016; Parker et al., 2016) than the effect sizes of QTLs for other complex traits.

Mouse and rat resources available to finely dissect the genetic basis of complex traits have been extensively reviewed (Peters et al., 2007; Flint, 2011; Flint and Eskin, 2012; Mott and Flint, 2013; Gonzales and Palmer, 2014; Williams and Williams, 2016). These reviews compare breeding schemes and relate them to genetic characteristics such as the total number of variants segregating, allele frequencies, rate of decay of linkage disequilibrium (a measure of how wide unrecombined intervals are), regions with low levels of polymorphism and specific analytic requirements. They also provide a structured discussion of the advantages and disadvantages of each population. By contrast, here we review genetic studies of complex neurobehavioural traits that have used these resources. We limit ourselves to resources with moderate to high levels of recombination (see Glossary, Box 1) for the reasons explained in the first section below, and finish by discussing the increasing relevance of outbred resources (see Glossary, Box 1).

**Box 1. Glossary of key terms**

**Complex trait:** A phenotype that varies as a result of multiple genetic and environmental effects and their interplay.

**X% confidence interval (CI):** Size of the genomic region that has X% chance to contain the variant responsible for the QTL.

**CRISPR/Cas:** Clustered regularly interspaced short palindromic repeats/CRISPR-associated proteins – a genetic engineering system based on components of a prokaryotic immune system that can be used to change single nucleotides or larger genomic fragments in any genome.

**Founder:** An inbred strain or outbred stock from which a resource is descended.

**Genetic mapping:** The process by which regions of the genome associated with phenotypic variation are identified.

**Genome-wide association study (GWAS)**: The genome-wide mapping of single-nucleotide polymorphisms (SNPs) associated with a particular trait across many individuals.

**Haplotype:** An unrecombined genomic segment inherited from one of the founders.

**Hardy–Weinberg equilibrium:** A variant is said to be in Hardy–Weinberg equilibrium when the frequencies of the corresponding genotypes are constant across generations. Unless a variant is subject to strong evolutionary forces (such as selection or meiotic drive), genotype frequencies will be approximately constant across generations at the time scales we are considering here (years or decades).

**Heritability:** The proportion of phenotypic variance in a population that is attributable to genetic effects.

**Inbred strain:** An inbred strain corresponds to animals that are genetically identical (clones). It is derived by sister-brother mating for many (>20 in mice) generations. In an inbred strain, all genetic loci are homozygous.

**LOD support interval:** The logarithm of the odds (LOD) ratio provides a measure of association between genotype and phenotype. The LOD will peak at a QTL and drop as distance to the QTL increases. In some circumstances the boundaries of a QTL can be defined based on the LOD profile and the distance it takes for it to drop by 1 unit, which gives 90% chance that thus-delimited QTL encompasses the causal variant. A 1.5 LOD drop interval corresponds to 95% confidence that the QTL encompasses the causal variant (Dupuis and Siegmund, 1999).

**Outbred stock:** Animals that are genetically diverse and unique.

**Quantitative complementation:** A method for testing the candidacy of a gene at a QTL. See Flint et al. (2005) for a description of this complicated test.

**Quantitative trait locus (QTL):** A locus in the genome found to be associated with variation in a quantitative phenotype, such as height, weight or a measure of anxiety (for example how much an animal freezes in response to a frightening stimulus). When the trait mapped is expression level of a gene, QTLs are called eQTLs (for expression QTLs) and classified as cis (cis-eQTL) when they are close to the gene and trans (trans-eQTL) when they are distant to it.

**Recombination event:** The exchange of genetic material between two homologous chromosomes during meiosis.

## Low levels of recombination in a population prevent gene identification

Gene identification typically starts with the mapping of QTLs and proceeds with integrative approaches, as reviewed recently by Moreno-Moral and Petretto (2016). A large number of QTLs for behaviour have been mapped in rodents [Mouse Genome Database, (Bult et al., 2016), www.informatics.jax.org; Rat Genome Database (Hayman et al., 2016; Shimoyama et al., 2014), http://rgd.mcw.edu/], but the genetic variants and genes that mediate the effects of these QTLs remain unclear (Flint et al., 2005; Flint and Mackay, 2009; Parker and Palmer, 2011). The main reason for this is that low levels of recombination exist in resources traditionally used for QTL mapping, namely F2 intercrosses and backcrosses (see Table 1 for a brief description of these populations).

**Table 1.**
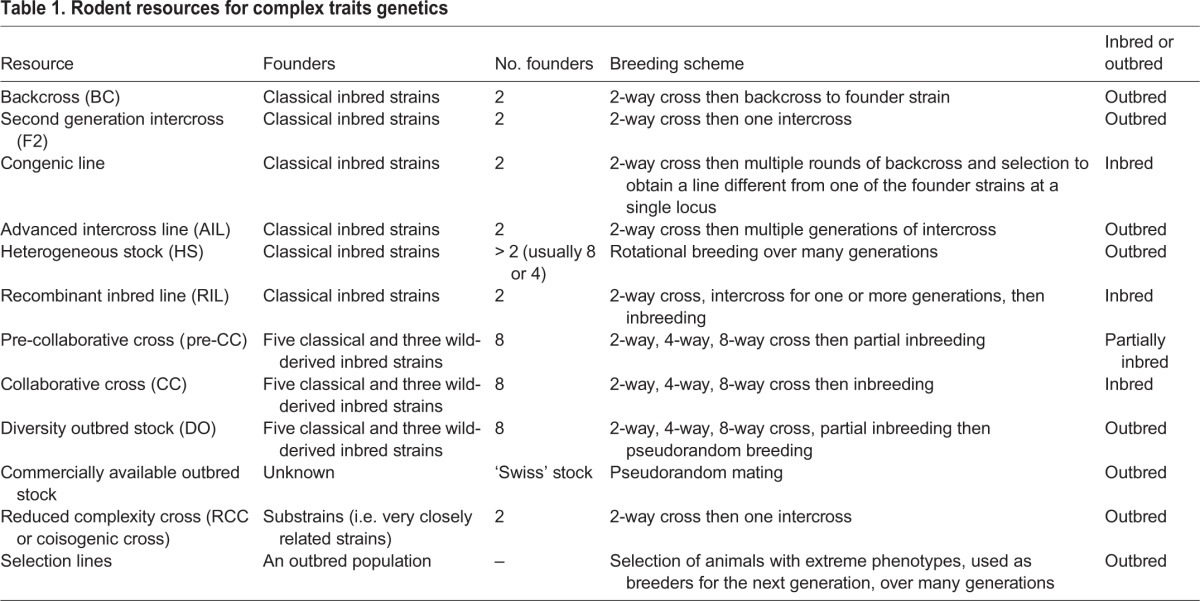
**Rodent resources for complex traits genetics**

**Fig. 1.**
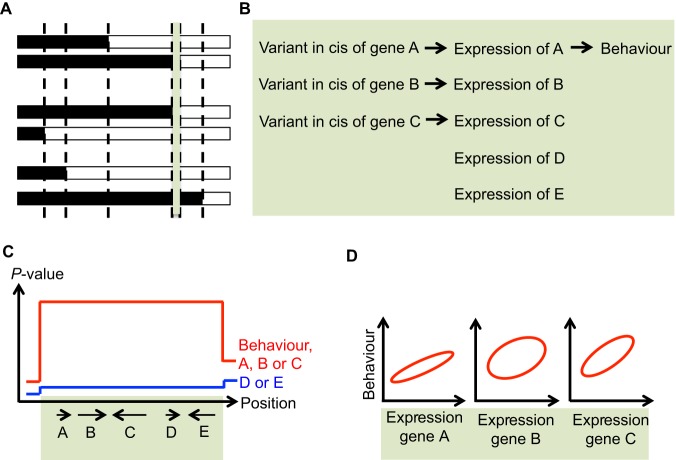
**Low levels of recombination preclude identification of genes associated with behavioural phenotypes.** (A) Large unrecombined genomic segments in F2 crosses. Three pairs of homologous chromosomes, from three individuals of an F2 cross, are represented. As each chromosome presents on average only one recombination event, large segments of DNA are unrecombined in F2 crosses (even with hundreds of individuals). One such segment is highlighted in green. (B) Example scenario for behavioural variation. Variation in behaviour arises from differences in expression of gene A driven by a variant in cis of the gene. Gene A lies in the unrecombined segment shown in green in panel A with many other genes, four of which (gene B to gene E) are presented in this scenario. Expression of genes B and C is controlled by variants in cis, while expression of genes D and E is not. In reality the proportion of genes with a cis-eQTL in any given tissue is about 10%. (C) Primary analysis: QTL mapping of the behaviour and of the genes encompassed by the behavioural QTL. The position along the chromosome is shown on the *x*-axis and spans the green unrecombined interval. Genes A to E are represented by arrows. The *y*-axis shows the significance of the association between genotype and trait (behaviour or gene expression level). The red curve shows the shape of the association curve for behaviour and genes A, B and C, the blue curve that for genes D and E. The shape of the red curve denotes a QTL for the behaviour and a cis-eQTL for each of genes A, B, C. As genes A, B and C have a cis-eQTL, they will be considered candidate causal genes for the behavioural QTL effect. (D) Gene-behaviour correlation analysis to further prioritise candidate genes. The ovals represent the spread of the data points (not shown); hence the tighter the oval, the stronger the correlation between the variables on the *x*- and *y*-axes. A correlation between expression of gene A and behaviour is likely to exist as a result of the causal path shown in panel B. As genes B and C also have cis-eQTLs and the underlying genotypes are perfectly correlated with the genotypes of the cis-eQTL for gene A (no recombination in the green interval), a correlation will likely exist between expression of genes B and behaviour and expression of gene C and behaviour. The strength of these correlations will depend on many parameters, including non-local genetic and non-genetic effects. Hence, prioritising genes A, B, C based on the strength (or significance) of the correlations is not straightforward, and is subject to caveats.

Each chromosome in an F2 population results, on average, from one recombination event between the two founder genomes, meaning that large segments of DNA are unrecombined (Glossary, Box 1 and Fig. 1A). As all genetic variants within an unrecombined segment are perfectly correlated, if one variant is associated with a given trait then the other variants in the segment will also be associated with that trait (Fig. 1D). As a result, F2 QTLs typically encompass a very large number of genetic variants and tens if not hundreds of genes, all of which are candidate mediators of the QTL effect.

In order to narrow down the list of candidates, common strategies include integrating sequence information and/or expression data with QTLs. Sequence information available for the parental strains of the cross (Keane et al., 2011; Yalcin et al., 2011; Baud et al., 2014b; Hermsen et al., 2015) enables identification of all the variants that segregate in the cross. Those variants whose effect is predicted to be deleterious for protein structure or function (e.g. using the variant effect predictor, http://www.ensembl.org/info/docs/tools/vep/index.html) can be deemed likely to mediate the QTL effect.

Another way to prioritise among the variants and genes at a QTL is to use expression data, usually obtained from mRNA present in a relevant tissue. Expression can be mapped to identify which of the genes at the QTL are regulated by a local variant i.e. genes with a cis-eQTL (see Glossary, Box 1). Such genes are usually prioritized over genes without a cis-eQTL as they could mediate the QTL effects through changes in gene expression (e.g. gene A in Fig. 1). The strength of the correlation between gene expression and behaviour is often used to select candidate genes (Fig. 1D), but there are caveats to this strategy (Doss et al., 2005; Mehrabian et al., 2005).

The strategies presented above have limited efficacy because typically, an F2 QTL encompasses a large number of deleterious variants (e.g. Parker et al., 2012) and about 10% of genes have a cis-eQTL in any tissue (Schadt et al., 2003). In addition, both strategies focus on protein-coding genes and make many assumptions, and therefore risk wrongly discarding the true genetic factors underlying the QTL. It is possible to alleviate these problems by mapping loci at higher resolution, thus reducing the number of candidate genes.

One way to obtain higher mapping resolution is to increase the sample size. However, obtaining adequate recombination levels with an F2 population would require phenotyping and genotyping of more than 10,000 animals (Mott and Flint, 2013). Breaking down one or a few intervals of interest into smaller unrecombined intervals by creating congenic lines (Table 1) is another option; however, such attempts have often seen the QTL disappear as it breaks down into two or more QTLs with smaller effects that cannot be detected (Flint and Mott, 2001; Legare et al., 2000). Finally, higher recombination levels can be achieved by crossing for multiple generations, as each chromosome will accumulate on average one meiotic recombination event each generation. Many breeding schemes exploit this process, and below, we discuss results obtained using the resulting populations.

## Mapping neurobehavioural traits in moderately to highly recombinant populations

Advanced intercross lines (AILs) (Darvasi and Soller, 1995) are generated from two inbred founders by multiple generations of intercrossing (Table 1). In laboratory rats, a seventh-generation (F7) AIL descended from the experimental autoimmune encephalomyelitis (EAE, a model for multiple sclerosis)-susceptible DA and EAE-resistant PVG.1AV1 inbred strains was used to fine-map QTLs previously detected in an F2 cross. Of 1068 phenotyped rats, 152 affected and 162 unaffected animals were genotyped and used to map QTLs that were 1.3 Mb, 3 Mb and 5.5 Mb wide (1 LOD support intervals; see Glossary, Box 1). Additional gene expression data, sequence data and prior knowledge were used to prioritise the genes at the QTL (Becanovic et al., 2006; Jagodic et al., 2004; Sheng et al., 2005).

A study of basal and methamphetamine-induced locomotor activity used 688 mice from a 34th-generation AIL descended from inbred strains SM/J and LG/J (Cheng et al., 2010). The authors report three genome-wide significant QTLs 0.5, 1.56 and 2.07 Mb wide (2 LOD support intervals), encompassing 1, 0 and more than 12 genes, respectively. *Csmd1* (CUB and Sushi multiple domains 1), which was identified in this study was knocked out for follow-up analyses but no effects on locomotor activity were detected (Distler et al., 2012). Another analysis of an eighth-generation AIL identified six QTLs ranging from 1.5 to 50 Mb in size, with a median of 15.6 Mb (1.8 LOD support intervals) (Parker et al., 2012). The studies highlighted here indicate that QTLs mapped in AILs can enable gene identification, provided enough recombination events have accumulated in the line over the generations.

Panels of recombinant inbred (RI) strains have traditionally been derived from inbreeding F2 animals (Table 1). In rats, two RI panels exist. The BXH/HXB panel was generated by reciprocal crossings of the spontaneously hypertensive rat (SHR/Ola) and the Brown Norway (BN-Lx/Cub) strains (Pravenec et al., 2004) and now contains 30 strains (Hubner et al., 2005). This panel has been used to map multiple behavioural traits, including startle, anxiety, locomotion, conditioned taste aversion, alcohol consumption and learning (Printz et al., 2003; Conti et al., 2004; Bielavská et al., 2002; Tabakoff et al., 2009; Vanderlinden et al., 2014; Stuchlik et al., 2012). Although QTLs mapped in these studies were too large to allow identification of candidate genes, the panel was successfully included in a broader genetic study that identified a determinant of cardiac hypertrophy and mitochondrial function (McDermott-Roe et al., 2011). The second panel is derived from LE/Stm and F344/Stm and consists of 34 strains. Mapping of 109 traits, including neurobehavioural traits, was performed in this panel, but QTL intervals were again too large (>20 Mb) to allow gene identification (Voigt et al., 2008).

In mice, larger RI panels exist: the LXS panel is descended from inbred long sleep (ILS) and inbred short sleep (ISS) strains, which were selected for their ethanol sensitivity, and consists of 77 strains. Using 60 of those and more than 10 mice per strain, Bennett et al. (2015) mapped the genetic determinants of acute functional tolerance to the hypnotic effects of alcohol and identified a 23 Mb QTL (90% Bayesian credible interval) that included 716 genes. Similarly, large QTLs were mapped in a study of hearing loss in the same panel (Noben-Trauth et al., 2010). The BXD panel, descended from the inbred strain C57BL6/J, which is the mouse reference strain, and another strain, DBA/2J, contains around 120 lines that are almost fully inbred and are available from the Jackson Laboratory, and another set of 30-40 that are being inbred by Williams, Lu and colleagues at the University of Tennessee Health Science Center (UTHSC) (Pandey and Williams, 2014). In this panel, 42 of the strains are derived from an F2 cross and the remaining are derived from F9 to F14 AILs, which improved the mapping resolution achievable with the panel (Peirce et al., 2004). It should be pointed out that very few studies make use of the full panel: many studies use only 30-40 strains and multiple replicates within each strain, resulting in very large QTLs (Putman et al., 2016; Harenza et al., 2014; Parsons et al., 2012; Ye et al., 2014; Young et al., 2016; Dickson et al., 2016).

Using 62 BXD strains, Carhuatanta et al*.* (2014) mapped five QTLs for fear and anxiety under chronic stress conditions. The size of the QTLs (1 LOD support intervals) ranged from 2.5 to 30.2 Mb with a median of 12.1 Mb. The narrowest interval encompassed only 15 genes, but no strong candidate gene was identified. Using 72 strains, Cook et al*.* (2015) mapped two QTLs 8.5 and 10 Mb in size (1.5 LOD support intervals) for anxiety-related traits following ethanol injection. Integrative approaches led the authors to prioritise four genes. Pandey and Williams (2014) used RNA expression data to show that the distance between the top cis-eQTL marker and the cognate gene could be as small as 0.6 Mb using 69 BXD strains. This demonstrates that the full BXD panel should enable causal genetic variants to be mapped very precisely. Using more strains would also improve the experimental power to detect QTLs. It is unclear, however, what the optimal number of strains and replicates is for a fixed number of mice. A very important advantage of the BXD panel is that a large amount of freely available phenotypic and molecular data that have been accumulated over the years on BXD strains and a web-based statistical analysis suite exists that can integrate these data to prioritise genes at QTLs (www.GeneNetwork.org). In this respect, the BXD panel is a unique resource.

**Fig. 2.**
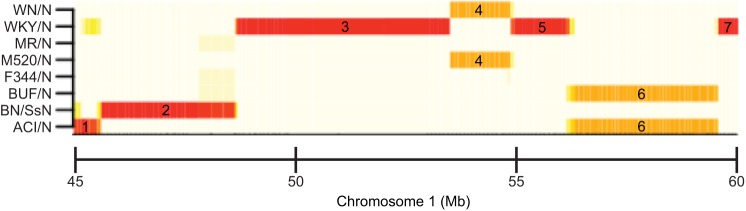
**Reconstruction of the chromosomes of an HS rat as a probabilistic mosaic of the eight founder genomes.** A 16 Mb region of chromosome 1 is represented (*x*-axis). The figure describes the probability that the pair of homologous chromosomes is descended from each of the eight founders (*y*-axis) using a colour code (white: 0; red: 2; yellow ∼1). Seven sub-segments (labelled 1-7) can be identified based on breaks in the colour pattern. The first sub-segment (45-45.5 Mb) has a probability of 2 (maximum probability) to be descended from the founder ACI/N; this means that we can be confident both homologous chromosomes are descended from that founder. Similarly, both chromosomes are descended from BN/SsN in the second segment and from WKY/N in the third segment. The fourth segment shows equal probability (probability of 1) to be descended from WN/N and M520/N; this can be interpreted either as one chromosome is descended from WN/N and the other from M520/N or those two founders are similar in that region and so we are unsure whether both chromosomes are descended from one or the other founder. Both chromosomes are descended from WKY/N in segments 5 and 7, and they are descended from BUF/N and/or ACI/N in segment 6. This fragmented pattern of genomic inheritance is referred to as ‘mosaic’. The probabilities can be used as input to the statistical models used to map QTLs.

AILs and RI panels exist that are descended not from two but four or eight inbred founder strains. They are respectively referred to as heterogeneous stocks (HS) (McClearn et al., 1970; Demarest et al., 2001; Hansen and Spuhler, 1984; Hitzemann et al., 2009; Iancu et al., 2010) and the collaborative cross (CC) (Churchill et al., 2004) (Table 1). HS are descended from the founder strains through usually more than 50 generations of outbreeding, which contributes to increasing mapping resolution. Mapping in HS and other populations descended from more than two inbred founders typically proceeds by reconstructing the chromosomes of the HS animals as mosaics of the founder genomes (Fig. 2) and mapping using haplotypes (Glossary, Box 1) rather than genotypes. QTL intervals obtained are typically 4 Mb wide (Rat Genome Sequencing and Mapping Consortium et al., 2013; Valdar et al., 2006a).

Because the complete sequence of the strains from which HS are derived is now available both in mice (Keane et al., 2011; Yalcin et al., 2011) and rats (Baud et al., 2014b; Hermsen et al., 2015), ‘merge analysis’ can be used to refine the mapping and prioritise variants and the genes affected for further study (Mott et al., 2000; Rat Genome Sequencing and Mapping Consortium et al., 2013; Valdar et al., 2006a). Briefly, merge analysis proceeds by identifying variants whose strain distribution pattern amongst the founder strains of the cross is consistent with the QTL effect. Importantly, such prioritisation is based solely on association statistics and therefore makes no assumption as to the mechanisms underlying the QTL (e.g. changes in gene expression, altered protein sequence). Anxiety and EAE-related traits have been investigated in the rat NIH HS (Hansen and Spuhler, 1984) and QTLs were identified where a single candidate gene was identified by merge analysis (Rat Genome Sequencing and Mapping Consortium et al., 2013; Baud et al., 2014a): *Ctnnd2* (catenin delta-2) is associated with conditioned anxiety and *Fam198b* is associated with weight loss as a result of EAE. A role for *Ctnnd2* in anxiety in mice is supported by altered behaviour of *Ctnnd2*-knockout mice in contextual fear conditioning paradigms (Israely et al., 2004) and an association between *CTNND2* and anxiety has been found in humans too (Nivard et al., 2014). *Ctnnd2* therefore seems to contribute to anxiety in multiple species (rats, mice, humans). At other QTLs mapped in the rat NIH-HS, additional information on the variants segregating at the QTL, namely their predicted effect on protein function, was used to identify candidate genes: MHC class II genes *RT1-Da* and *RT1-Bb* were identified as potential contributors to EAE-associated weight loss. In mice, independent mapping of emotionality in the Boulder and the Northport HS (Talbot et al., 1999; Valdar et al., 2006a) identified the same 2 Mb region around *Rgs2*, a gene that was proven to underlie a QTL for emotionality in commercially available outbred mice (described below, Yalcin et al., 2004). Fine-mapping of anxiety-related traits in the Northport HS together with analysis of functional gene annotations and testing of a conditional mutant mice provided evidence that actin filament depolymerisation and expression of Cofilin-1 (*Cfl1*) in the ventral hippocampus may mediate anxiety (Goodson et al., 2012).

The rat NIH-HS was used to derive replicated high- and low-alcohol-drinking lines through bi-directional selection (Foroud et al., 2000) (Table 1). Subsequent mapping of alcohol preference identified QTLs that were too large for gene identification but, combined with an analysis of signatures of selection, the selection lines pointed to specific genes (*Grin2a*, *Cyp4f18*, *Myo9b*, *Pgls* and *Fam129c*) (Lo et al., 2016), illustrating the potential of this combined approach.

The CC is a panel of recombinant inbred strains descended from eight inbred founders including three wild-derived inbred strains, which ensure a very high level of genetic diversity and variation in almost any trait of interest (Churchill et al., 2004). Interestingly, the wild-derived strains contribute genetic variants that cause anomalous behaviour in CC mice (in comparison with classical laboratory mice) with practical consequences, thus care is required when interpreting behavioural data (Chesler, 2014). Published studies have so far mostly used pre-CC mice, i.e. mice from partially (∼75%) inbred lines in the CC breeding colonies (Table 1). Using pre-CC mice, high mapping resolution was achieved across a range of phenotypes and molecular traits. QTLs for reproductive, behavioural, physiological and morphological traits were 4 Mb wide on average (1.5 LOD support intervals) (Philip et al., 2011). Philip et al. (2011) reported that the QTL interval detected for ‘activity after sleep deprivation’ harbours only three positional candidates: a microRNA and two genes: *Ntm* (neurotrimin), a neural cell-adhesion molecule that plays a role in brain development and *Snx19* (sorting nexin 19), which possesses a regulator of G protein-coupled receptor signalling (RGS) domain. The same authors identified a QTL interval for thermal nociception comprising just six genes: *Slit2*, *Pacrgl*, *Gpr125*, *Dhx15*, *Sod3* and *Kcnip4*.

Large pre-CC studies typically used many lines and one or few mice per line, suggesting that more lines and fewer replicates per line may be the best design for genetic mapping. Breeding of pre-CC mice is difficult and many lines have gone extinct (Threadgill et al., 2011; Chesler et al., 2008). A subset of 75 (as of time of writing) extant lines have reached a sufficient degree of inbreeding and meet criteria to be distributed to all investigators [available lines are updated at www.csbio.unc.edu/CCstatus/index.py?run=AvailableLines; see also Morgan and Welsh (2015) for informatics resources available to analyse CC data]. As this number is much lower than the number of lines used in pre-CC studies (∼250 in pre-CC studies) and because CC lines have different genetic characteristics compared with pre-CC lines, the potential of CC mice needs evaluating. CC lines that are available to all investigators have been used in a very limited number of published studies to date and these studies used only a subset of all currently available lines (e.g. 16 in Mao et al., 2015). Thus, the potential of currently available CC lines to allow identification of genes associated with behaviour and other complex traits is unclear.

The CC panel – and indeed any recombinant inbred panel – can further be used by intercrossing CC lines (Graham et al., 2015; Rasmussen et al., 2014) or crossing CC lines to an inbred strain with a genetic variant of interest to identify modifiers of the variant (Chitsazan et al., 2016).

An outbred population of mice called diversity outbred (DO) stock (Svenson et al., 2012; Churchill et al., 2012) was also derived from pre-CC lines (Table 1). A website has been set up to host DO data and support their analysis (do.jax.org; see also Morgan and Welsh, 2015; Gatti et al., 2014). A total of 38 behavioural measures related to activity, anxiety and response to novelty were mapped in 283 DO mice from generations 4 and 5. Five significant QTLs were identified, with 1.5 LOD support intervals ranging from 1 to 7 Mb (Logan et al., 2013). A QTL for climbing frequency during the tail suspension test (a measure described by the authors as one of wildness rather than depressed mood, which the test is traditionally used to measure) encompassed only five genes. Fine-mapping by identifying those variants at the QTL that have a strain distribution pattern that is consistent with the QTL effect in the founder strains (a similar approach to merge analysis) failed for this and some of the other QTLs, because variants with the appropriate strain distribution pattern were found to populate the entire QTL region (Logan et al., 2013). This situation is expected to be common with DO QTLs as a result of wild-derived founder strains being very different from each other and from the five classical inbred founder strains, and because wild-derived alleles drive most of the QTLs. As DO and CC share the same founders, this situation is also common in CC QTLs (Durrant et al., 2011). As mentioned and illustrated in Logan et al. (2013), wild-derived genetic variants also cause DO mice (like CC mice) to behave ‘inappropriately’ on certain tests (e.g. climbing in the tail suspension test), thus warranting care in the analysis and interpretation of the corresponding data.

Although both CC and DO were designed to present as much genetic variation as possible, an opposite strategy led to the development of reduced complexity crosses (RCC, also called coisogenic crosses). RCC are crosses of two closely related strains, typically two strains that are descended from a single inbred strain through independent evolution in different laboratories (Table 1). Kumar et al. (2013) observed differences in cocaine response between C57BL/6J, a C57BL/6 strain established and maintained at the Jackson Laboratory since 1948, and C57BL/6N, a C57BL/6 strain established at NIH in 1951. Subsequent mapping in an F2 cross identified a QTL that accounted for 61% of the genetic variance (i.e. the phenotypic variation of genetic origin). The QTL interval was 22 Mb wide, which in any other cross would have precluded identification of candidate genes. However, because there is very little genetic variation between the two founder strains, a single genetic variant (not gene) affecting the protein sequence within the QTL interval was suspected to cause the QTL effect. This variant encodes a serine-to-phenylalanine missense mutation in *Cyfip2* (cytoplasmic FMRP-interacting protein 2), a gene implicated in Fragile-X mental retardation.

Kumar et al. (2013) followed up on this candidate gene by measuring the stability of the corresponding protein in the founders C57BL/6J and C57BL/6N, and by behavioural, histological and electrophysiological profiling of a *Cyfip2* mouse knockout. All analyses supported a role for *Cyfip2* in the response to cocaine exposure. This study illustrates how crosses where only a few genetic variants segregate can solve the recurrent problem of translating QTLs into information about causal genes and variants. Importantly, this study also shows that in RCC-based studies, the size of QTL intervals is not a good indication of the potential to identify causal variants and genes (Williams and Williams, 2016), as it is limited by genotyping density rather than recombination levels.

Additional phenotypes including behaviours have been shown to vary across C57BL/6 substrains (Simon et al., 2013; Khisti et al., 2006; Mulligan et al., 2008; Kirkpatrick and Bryant, 2014) and are amenable to genetic mapping. Substrains also exist in the laboratory rat and the genetic variants that segregate between some of them have been catalogued (Atanur et al., 2013; Hermsen et al., 2015), allowing genetic study of phenotypic differences (Zhang-James et al., 2013).

The last type of resource we will review is commercially available outbred rats and mice, which refers to animals that have been bred by commercial vendors for decades, primarily for use in pharmacological studies (Table 1). These populations are typically maintained using an outbreeding regime in order to preserve genetic diversity. For this reason, they should never be called ‘strains’ as a strain is the result of many generations (usually 20) of inbreeding. Moreover, a review of commercially available mice by Yalcin et al. (2010) highlighted important genetic differences between colonies of the same stock, where colony refers to ‘a population of mice maintained as a mating population at a single location’, and stock refers to ‘a collection of colonies that are given the same stock designation by the breeders’. Thus, colony is the unit of interest for genetic studies using commercially available outbred mice, and the same is likely to be true for rats. Yalcin et al. (2010) investigated genetic characteristics that are important for genetic mapping (in particular, rate of linkage disequilibrium decay and minor allele frequencies) of 66 available colonies, providing valuable information to those wishing to choose a colony for genetic mapping. Prior to this study, 729 mice from the HsdOla:MF1-UK colony (nomenclature according to Yalcin and Flint, 2012) had been used to fine-map a chromosome-1 QTL for anxiety in mice (Yalcin et al., 2004). The study led to the identification of a regulator of G-protein signalling, *Rgs2*, as a strong positional candidate, and confirmed that it accounted for some of the QTL effect by quantitative complementation (Glossary, Box 1).

In 2016, two studies were published that investigated the genetic basis of a variety of complex traits, including many neurobehavioural traits, in commercially available outbred mice from the Crl:CFW(SW)-US_P08 (CFW) colony (Nicod et al., 2016; Parker et al., 2016). The size of the 95% CI for the 255 QTLs mapped by Nicod et al. (2016) using ∼1800 mice ranged from 0.01 to 7.33 Mb, with a mean at 1.50 Mb and 43% of intervals smaller than 1 Mb (the size of CIs was not reported by Parker et al., 2016). It constitutes the highest mapping resolution achieved genome-wide in any rodent resource thus far. In the study by Nicod et al. (2016), 7 QTLs for neurobehavioural traits encompassed a single gene, thus identifying 7 very strong positional candidates: (1) Glutamate receptor metabotropic 7 (*Grm7*) was associated with total distance travelled in elevated plus maze (see Ellenbroek and Youn, 2016 for a description of this test) and a role for *Grm7* in locomotor activity in a new environment is supported by evidence from a mouse knockout (Cryan et al., 2003). (2) Unc-13 homolog C (*Unc13c*) was associated with the number of long (>1 min) sleep episodes, and there is also evidence for differential expression of the human orthologue in individuals with poor sleep quality (Reddy et al., 2014). (3) *Met* and (4) *Rtkn2* were associated with startle pulse reactivity; (5) *Ppargc1a* with number of long (>1 min) sleep episodes; (6) *Adarb2* with basal home cage activity and (7) *Pcdh17* with total distance travelled in elevated plus maze, although there was no independent evidence for a role of these genes in these phenotypes.

In addition to collecting multiple behavioural phenotypes related to conditioned fear, anxiety-like behaviour, methamphetamine sensitivity and prepulse inhibition on ∼900 mice, Parker et al. (2016) measured gene expression in a subset of the mice in the hippocampus (*n*=79), striatum (*n*=55) and prefrontal cortex (*n*=54). Integrating QTLs for behavioural and gene expression traits, they prioritised those genes at QTLs that had a cis-eQTL in a relevant brain region and whose expression was correlated with the behaviour. *Azi2* (5-azacytidine-induced gene 2) was the best candidate gene at a QTL for methamphetamine sensitivity, and *Zmynd11* (zinc finger, MYND domain-containing 11) at a QTL for anxiety-like behaviour. *Zmynd11* has independently been suggested to be involved in various psychiatric disorders (Coe et al., 2014). Commercially available outbred rats also exist, but the genetic characteristics of the different colonies have not yet been assessed, nor have such rats been used in mapping studies.

We recapitulate in Table S1 the 54 significant QTLs reported in the studies reviewed above and that have led to the identification of 1 to 5 positional candidate genes. This table demonstrates that the genetic determinants of behaviour can now be mapped with high precision both in rats and mice. Importantly, we have not reported in Table S1 genes cloned using congenic strains. Those would only strengthen our point, however, which is that the time is over when high numbers of positional candidate genes at QTLs were the limiting factor for the usefulness of rodent models in genetic studies. What seems to be the limiting factor is our ability to follow up on narrow QTLs with experimental validation and functional characterization, as discussed below.

## Validation of QTLs: challenges and opportunities

Candidate genes have been followed up for only three out of 54 QTLs (for a fourth QTL, there was prior evidence confirming a role of the candidate gene *Ctnnd2*). The first case is that of *Rgs2*, a gene associated with anxiety in the mouse Northport heterogeneous stock. Its contribution to the QTL was confirmed by quantitative complementation (Yalcin et al., 2004). The second case is *Csmd1*, a gene associated with locomotor activity in a 34th-generation AIL (Cheng et al., 2010). In this case, no effect of the gene on locomotor activity was observed in the corresponding knockout model (Distler et al., 2012). The third example is *Cyfip2*, a gene associated with cocaine response in a RCC between the C57BL/6J and C57BL/6N substrains (Kumar et al., 2013). Follow-up included demonstrating differential stability of CYFIP2 between the founder strains, and histological, electrophysiological and behavioural testing of a *Cyfip2* knockout.

Why are QTLs so difficult to follow up on? Firstly, there is ever increasing evidence, mostly from human genetics studies, that the mechanisms of genetic control are not straightforward. In line with this, 43% of trait-associated SNPs in humans lie in intergenic regions and 45% lie in introns (Hindorff et al., 2009); intronic variants have been shown to regulate distant genes in some instances (Rask-Andersen et al., 2015); finally, there is also mounting evidence that QTLs often arise from multiple variants and possibly multiple genes (Rat Genome Sequencing and Mapping Consortium et al., 2013; Allen et al., 2010). As a result, investigators may have limited confidence in the variants or genes they identify as candidates at QTLs.

A second difficulty lies in creating a genetically engineered knockout model in order to validate a candidate gene. Until recently, knockout models were available for only a few genes, and creating a knockout model was very time- and resource consuming. However, things are changing: first, more and more knockouts are becoming available through a very large international consortium – the International Mouse Phenotyping Consortium (http://www.mousephenotype.org/); secondly, the recent advent of the CRISPR/Cas technology for genome editing (Glossary, Box 1) has made it possible for individual laboratories to easily create their own, personalised knockout model (Barrangou, 2014). We refer the reader to three reviews on CRISPR/Cas technology, which include a comparison with ZFN and TALEN technologies (Gaj et al., 2013; Kim and Kim, 2014; Sander and Joung, 2014). In brief, the simplicity and high efficiency of the CRISPR/Cas system makes it a very attractive alternative to traditional knockout procedures.

In the future, it is possible that knockouts will not be the go-to models for following up on QTLs. Indeed, variants underlying QTLs have subtle effects that loss-of-function models poorly recapitulate, and variants associated with complex traits have been shown to often sit outside the protein-coding regions. Thus, it may be more appropriate to validate variants rather than genes. CRISPR/Cas technology permits replacement of specific single nucleotides and addition or deletion of specific sequences. For example, Yoshimi et al. (2014) showed that they could revert each of three mutations of F344 rats (albino, non-agouti and hooded). Thus, CRISPR/Cas opens new avenues for follow up of QTLs through variant editing. Unfortunately, even the highest mapping resolution achieved to this day leaves tens if not hundreds of variants segregating at most QTLs. To narrow down the list of candidate variants to a tractable number (10, 20 maybe for CRISPR), one can use additional information regarding the position of the variants (e.g. lying in a promoter or at a splice site), their predicted effect on protein structure (e.g. predicted to affect binding with DNA), functional evidence from the mouse ENCODE project (www.mouseencode.org/), or from analysis of evolutionary constraints, for example.

Finally, an added difficulty of validating a QTL using a genetically engineered model is the genetic background on which the mutant is created. The same mutation may have observable effects in one background but not in another (Holmes et al., 2003) and it may even have opposing effects in different backgrounds (Sittig et al., 2016) Therefore, when the goal is to validate a QTL, a mutant should ideally be created on the background in which the QTL was detected. Unfortunately, in all mapping populations except for congenic lines, genetic variants exist not only in the QTL interval but everywhere in the genome; this means that each animal or strain of a mapping population represents a unique genetic background and there is not a single background on which the QTL should be placed. One solution is to place QTL alleles on multiple genetic backgrounds representative of the original population. For example, the same mutation could be introduced in multiple DO mice and mutant DO mice compared with non-modified DO mice. This expensive validation strategy will maximize the investigators' chances to replicate the QTL effect, and will have the added benefit of increasing the generalisability of the results to multiple genetic backgrounds – an important first step towards applying the information to humans (Sittig et al., 2016). The efficiency of the CRISPR/Cas technology in seemingly any background is yet another major advantage of this technology, which truly seems to hold the key to the future of animal model-based investigation of complex traits.

## Inbred versus outbred resources

In light of the success of outbred resources in precisely mapping phenotypic variation, we wish to discuss what has been seen as unique advantages of inbred resources. Inbred resources include RI panels derived from two or more progenitors and combinations of inbred and RI strains [e.g. the hybrid mouse diversity panel, HMDP (Bennett et al., 2010)]; outbred resources include AIL, HS, DO and commercially available outbred stocks. Traditionally, the main selling points of inbred resources were threefold. First, most inbred strains have already been genotyped and need not be genotyped again, whereas outbred animals are genetically unique and thus need to be genotyped in each experiment. Recently, next-generation sequencing (NGS) has been used to genotype commercially available outbred mice, using two different approaches (Davies et al., 2016; Parker et al., 2016), showing that NGS is an attractive alternative to microarrays for genotyping outbred populations.

Second, multiple measurements can be made on the same genotypes (using multiple animals of the same inbred strain), thus reducing non-genetic variation and facilitating detection of genetic associations. However, when inbred panels are large enough (e.g. BXD RI panel), using all strains and only one animal per strain might be more powerful than using fewer strains and multiple animals per strain (Fig. 1B in Andreux et al., 2012). Using one animal per strain effectively means using an outbred sample.

The third advantage is that the same genotypes can be used in multiple experiments. This permits accumulation of phenotypic data over the years, which facilitates systems genetics approaches. The best example of this is the accumulation of more than 5000 organismal phenotypes, mRNA expression in ∼33 tissues, microRNA and protein expression data, as well as metabolomics data in the BXD RI panel (www.genenetwork.org). The availability of multiple animals with the same genotypes has also facilitated studies of gene by environment (G×E) interactions and sex-specific genetic effects (G×S) (Peirce et al., 1998). Last but not least, this panel has been used to evaluate the robustness of positive findings through replication studies.

However, it is possible to tackle these goals using outbred populations, as illustrated by the following studies. Krohn et al. (2014) identified G×S effects in outbred HS mice, showing that such effects were widespread but relatively small. French et al*.* (2015) replicated benzene-induced genotoxicity in one study of two cohorts of DO mice, illustrating the concept of replication for different sets of genotypes. They did not go as far as mapping QTLs in the two cohorts however, but instead combined them for QTL mapping (presumably because of sample size considerations). In theory, genotype to phenotype associations involving variants in Hardy–Weinberg equilibrium (see Glossary, Box 1) could be investigated in multiple samples collected in different generations of an outbred population, as allelic frequencies are expected to remain constant. However, there is a caveat in that other variants that are not in Hardy–Weinberg equilibrium might cause differences in genetic background and interact with the evaluated variant, modifying its effect. Thus, the potential of outbred populations for replication studies and to investigate G×S and G×E interactions remains to be fully exploited.

## Conclusions

Multiple highly recombinant rat and mouse resources now allow precise mapping of phenotypic variation. However, experimental validation and functional characterisation of candidate genes remain major obstacles in the way of identifying causal genes and pathways. Both steps are crucial to turn QTLs into biological understanding of the mechanisms underlying behaviour. The advent of the CRISPR/Cas technology holds great promises in this regard, yet important experimental parameters such as the genetic background on which mutants are generated need to be considered carefully.

It is important to stress that many of the resources highlighted here are not fixed – they are evolving, be it by addition of new RI strains (e.g. BXD panel), further inbreeding of current lines (e.g. CC) or further outbreeding of current population (e.g. DO, HS). Furthermore, new resources can easily be created from existing ones (e.g. RIX), and resources can be combined (Bennett et al., 2010; Furlotte et al., 2012). Thus, rodent resources can only improve in the future.
